# Comparison of the sequences and expression levels of genes related to follicular development and atresia between prolific and nonprolific goat breeds

**DOI:** 10.1002/vms3.225

**Published:** 2019-11-28

**Authors:** Xiang‐Dong Zi, Liang Hu, Jian‐Yuan Lu, Shuang Liu, Yu‐Cai Zheng

**Affiliations:** ^1^ Key‐Laboratory for Animal Science of State Ethnic Affairs Commission Southwest Minzu University Chengdu China; ^2^ Key Laboratory of Conservation & Utilization of Qinghai‐Tibetan Plateau Animal Genetic Resources of Ministry of Education Southwest Minzu University Chengdu China

**Keywords:** cloning, expression, goats, hormonal concentration, prolificacy

## Abstract

This study investigated the variations of the nucleotide sequences and ovarian expression levels of genes related to follicular development and atresia in prolific Jintang black goats and nonprolific Tibetan goats. Eight genes, *FSHB*, *LHB*, *FSHR*, *LHCGR*, *ESR2*, *B4GANT2*, *BCL2* and *BAX*, were examined using reverse transcription‐polymerase chain reaction and quantitative real‐time PCR. The results showed that the nucleotide and deduced amino acid sequences of the *LHB* and *BAX* genes were not different, but there was one base change in the *FSHR* genes between the two breeds. There was one base change in the *FSHB* gene, which resulted in one amino acid substitution; there were nine base changes in the *LHCGR* gene, which resulted in five amino acid substitutions; and there were six base changes in the *B4GANT2* gene, which resulted in four amino acid substitutions. The expression levels of the *FSHR*, *LHCGR*, *ESR2*, *B4GANT2*, *BCL2* and *BAX* genes in the ovaries were not different between the two breeds. The plasma concentrations of FSH were not different, but the plasma concentrations of LH, P_4_ and E_2_ were lower in prolific Jintang black goats than in nonprolific Tibetan goats (*P* ˂ 0.05) at 40 hr after removal of the Controlled Internal Drug Release Devices. These results provide some foundations elucidating the endocrine and molecular mechanisms controlling ovulation rate in goats, but these need to be further verified.

## Graphical abstract

Plasma concentrations of LH, P4 and E2 were lower in the prolific goat breed. FSH concentration was not different between prolific and non‐prolific goat breeds. Nine base changes in LHR resulted in five amino acid substitutions in prolific breed, and six base changes in B4GANT2 resulted in four amino acid substitutions in prolific breed.

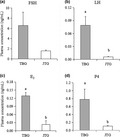

## 1 INTRODUCTION

Ovulation rate is the most important determinant of litter size in sheep and goats (Notter, 2012). At the early stages of follicular growth, the gonadotrophins follicle stimulating hormone (FSH) and luteinizing hormone (LH) do not appear to be definite prerequisites for follicular development, but at the later stages, FSH and LH have a central role in follicle growth and maturation through their interactions with receptors (FSHR and LHCGR) in granulosa cells (Hunter, Robinson, Mann, & Webb, 2004; Webb et al., 2003). Follicle recruitment and development leading to ovulation can be increased by the manipulation of these hormonal inputs (Mendes et al., 2018). However, in most studies, no clear differences in the plasma concentrations of LH and FSH between sheep and goat breeds with different ovulation rates have been found (Abdennebi et al., 1999; Adams, Quirke, Hanrahan, Adams, & Watson, 1988; Bartlewski et al., 1999; Bindon, Blanc, Pelletier, Terqui, & Thimonier, 1979; Bindon et al., 1985; Cahill et al., 1981; Cui et al., 2009; Drouilhet et al., 2010; Lahlou‐Kassi, Schams, & Glatzel, 1984; McNatty, Gibb, Dobson, & Thurley, 1981; Webb & England, 1982). Similarly, the relationship between the expression levels of FSHR and LHCGR during the periovulatory period and rate of ovulation is ambiguous (Abdennebi et al., 1999; Cui et al., 2009; Drouilhet et al., 2010; Regan et al., 2015; Zi, Huang, Wang, & Lu, 2013). More recently, Drouilhet et al. (2013) reported that the highly prolific Lacaune sheep is associated with an ectopic expression of the beta‐1, 4‐N‐acetyl‐galactosaminyl transferase 2 (*B4GALNT2*) gene within the ovary.

It is possible that prolific and nonprolific breeds are characterized by differences in follicular atresia. More than 99.5% of the ovarian follicles that are present at birth do not reach ovulation; therefore, the most common fate of follicles is to undergo atresia (Aitken, Findlay, Hutt, & Kerr, 2011; Kaipia & Hsueh, 1997; Tilly, Kowalski, Johnson, & Hsueh, 1991). Accumulating evidence shows that ovarian follicle atresia is mediated by apoptosis (Markstrom, Svensson, Shao, Svanberg, & Billig, 2002; Rolaki, Drakakis, Millingos, Loutradis, & Makrigiannakis, 2005). BAX and BCL2 have been proposed as key mediators in controlling apoptosis in the ovaries of many mammals (Almog et al., 2001; Gursoy, Ergin, Başaloglu, Koca, & Seyrek, 2008; Jensen, Willis, Albamonte, Espinosa, & Vitullo, 2006; Kugu et al., 1998; Poljicanin et al., 2013; Sai et al., 2011; Yang & Rajamahendran, 2000; Zhang et al., 2015) and avian (Van Nassauw, Tao, & Harrisson, 1999).

In general, the mechanism that controls ovulation rate has not been studied in goats as extensively as that in sheep. The prolificacy‐associated markers of sheep in the *BMPR1B*, *GDF9* and *BMP15* genes have not been detected in Chinese goat breeds (He, Ma, Liu, Zhang, & Li, 2010; Hua, Chen, Ai, & Yang, 2008) or Indian goat breeds (Ahlawat, Sharma, & Maitra, 2013), although Polley et al. (2009) found that in Indian Black Bengal goats, the *BMPR1B* gene was polymorphic. Some novel SNPs in these three candidate genes for prolificacy were genotyped in Indian breeds, but they do not contribute to the reproductive capability (Ahlawat et al., 2016). The Jintang black goat (JTG) is a local Chinese breed that is famous for its high fecundity, with an average kidding rate of 250%. The Tibetan goat (TBG, *Capra circus*) is a single‐birth breed that is characterized by its adaptation to cold, hypoxic ecological conditions in the Qinghai‐Tibet Plateau (Editorial Committee of Animal Genetic Resources in China, 2011). Therefore, the objectives of this study were to examine if there are any variations in the concentrations of FSH, LH, estrogen (E_2_) and progesterone (P_4_), in the sequences and mRNA expression levels of the FSH beta subunit (*FSHB*), LH beta subunit (*LHB*), *FSHR*, *LHCGR*, estrogen receptor beta *(ESR2)*, *B4GALNT2*, *BCL2* and *BAX* genes, and plasma concentrations of FSH, LH, estradiol (E_2_) and progesterone (P_4_) between these two goat breeds.

## 2 MATERIALS AND METHODS

### 2.1 Ethics statement

All of the experiments were performed according to the Regulations for the Administration of Affairs Concerning Experimental Animals (Ministry of Science and Technology, China; revised in August 2011) and approved by the Institutional Animal Care and Use Committee of Southwest Minzu University, Chengdu, China.

### 2.2 Animals and sample collection

All the selected animals were of the same age (4‐years‐old) and parity (four parities), and estrus was synchronized to the same stage. The prolific JTG (*n* = 5) that were chosen were those with a history of successive multiple births (≥ triplet births, mean prolificacy = 3.80 ± 0.45), while the nonprolific TBG (*n* = 5) that were chosen were those with a history of successive single births. The goats were synchronized using Controlled Internal Drug Release Devices (CIDR) (Eazi‐Breed CIDR, InterAg, Hamilton, New Zealand) for 13 days. At 24 hr before the CIDR removal, all of the goats were treated with 3.75 mg of the PGF_2α_ analogue luprostiol (0.5 ml Prosolvin, Intervet Ireland Ltd., Dublin, Ireland). Jugular venous blood samples were collected by venipuncture at 40 hr after CIDR removal, and the goats were then immediately slaughtered. The intact ovaries and anterior pituitaries were collected 5 min after slaughter and frozen in liquid nitrogen. They were then stored at −80°C for further RNA extraction. All of the blood samples were collected into EDTA tubes and then centrifuged for 20 min at 400*g*. Plasma samples were stored at −20°C until the hormone assays.

### 2.3 Hormonal assays

Plasma concentrations of FSH, LH, E_2_ and P_4_ were measured at 40 hr after CIDR removal by ELISA using commercial kits designed for goats (NanJing SenBeiJia Biotechnology Co., Ltd., China). The intra‐ and inter‐assay CVs for all of the ELISA kits were less than 9% and 11%, respectively. The sensitivities of the FSH, LH, E_2_ and P_4_ assays were 0.02 ng/ml, 0.05 ng/ml, 0.02 ng/ml and 0.05 ng/ml, respectively.

### 2.4 RNA isolation and reverse transcription‐polymerase chain reaction (RT‐PCR)

Total RNA was extracted from intact ovaries and anterior pituitaries with RNAprep pure Tissue Kit (Tiangen Biotech, Beijing) following the manufacturer's instructions. The samples were quantified using a spectrophotometer (Eppendorf, Germany), and RNA integrity was evaluated on a 1% (w/v) denaturing agarose gel. All samples were stored at −80°C until cDNA synthesis. Reverse transcription was performed using TaKaRa RNA PCR Kit (AMV) (TaKaRa, Dalian, China), according to the procedure supplied by the manufacturer. The reaction was incubated for 30 min at 42°C, inactivated by heating the reaction to 98°C for 5 min, and stored at −20°C.

### 2.5 Gene Cloning

All of the primers (Table 1) were designed using Beacon designer 7 and were synthesized by Invitrogen (Shanghai, China). The regions of cDNA were amplified using 0.5 μL anterior pituitary cDNA for the *FSHB* and *LHB* genes and 0.5 μL ovarian cDNA for the *FSHR, LHCGR, B4GALNT2, BCL2* and *BAX* genes of the two goat breeds. The PCR was performed in the presence of 12.5 μ*L* 2 × Long Taq PCR MasterMix (Tiangen Biotech, Beijing) and 10 μM of the forward and reverse primers in a final volume of 25 μL. The optimal PCR conditions (Table 1) were determined for the amplifications. The expected length of the PCR products included the complete coding sequences so that the complete coding sequences of these seven genes could be obtained by an analysis of the expected nucleotide sequence. The complete coding sequences of these genes were directly amplified. The PCR products were analyzed by electrophoresis in 1% agarose gels.

**Table 1:** Primer pairs used for gene cloning and optimal PCR condition for gene cloning

Genes	Primer sequence (5′→3′)	Accession no.	Product size (bp)	Cycle profile	Cycles
*FSHB*	F: GATGAAGTCCGTCCAGTT R: GCTGCTGCTCTTTATTCTC	NM_001009798.1	402	94°C/45 s, 52.4℃/30 s, 72℃/60 s	30
*FSHR*	F: CGGGGTGGATGGATAAGTAAAC R: ATGAAGTATGTGGAAGTGCTCTG	NM_174061.1	2,208	94°C/45 s, 51.5°C/45 s, 72°C/120 s	35
*LHB*	F: GATGGAGATGCTCCAGGGAC R: GAAGTCTTTATTGGGAAGGGAG	NM_001009380.1	510	94°C/45 s, 56.3°C/30 s, 72°C/60 s	30
*LHCGR*	F1: ATGGGACGGCCGTCCCTCGC R1: CTTGGTATGGTGGTTATGTG	NM_174381.1	503	94°C/30 s, 56.3°C/45 s, 72°C/45 s	30
	F2: ACCCGACTGTCACTCACCTAT R2: TTGCCTGATGTGCCTAACAC	NM_174381.1	1966	94°C/45 s, 54°C/45 s, 72°C/140 s	35
*B4GALNT2*	F1: ATTCGTGATGACTTCGTTCG R1: CACGGTCAAGTCTGGGTAAT	KC175557	883	94°C/40 s, 51°C/40 s, 72°C/60 s	35
	F2:GACCATCCGCTTTCCTGTTA R2:AGTGATTCCCAAATGGTAAGA	KC175557	940	94°C/40 s, 53°C/40 s, 72°C/60 s	35
BCL−2	F:ATGGCGCACGCGGGGGGAACA R:TCACTTATGGCCCAGATAGG	NM_001166486.1	690	94°C/40 s, 51.7°C/40 s, 72°C/140 s	35
BAX	F:ATGGACGGGTCCGGGGAGCAA R:TCAGCCCATCTTCTTCCAGA	NM_173894.1	579	94°C/40 s, 63.1°C/40 s, 72°C/40 s	35

Abbreviations: F, Forward primer; R, Reverse primer.

### 2.6 Nucleotide and amino acid sequence analysis

Following agarose gel electrophoresis, the purified PCR product was ligated into a p‐GEM‐T vector (Qiagen, Germany), and the recombinant plasmids were identified from the transformed bacterial colonies using standard techniques (Sambrook, 2001). The plasmid DNA was isolated using a plasmid isolation kit (Promega, USA). After using the appropriate restriction enzymes, the clones were sequenced by an automated sequencer (Perkin‐Elmer, Foster City, CA) using Sanger's dideoxy chain termination method by Shanghai Invitrogen Biotechnology Ltd. Co. (Shanghai, China). All of the animals were sequenced. The sequence that was obtained was subjected to BLAST analysis (://www.ncbi.nlm.nih.gov/BLAST) to verify that the sequence was of these target genes. Nucleotide sequence identity was performed using the Clustal program of MegAlign (Lasergene software, DNASTAR). An alignment of the deduced amino acid sequence was also performed by the Clustal multiple sequence alignment program.

### 2.7 Quantitative real‐time PCR (QPCR)

The qPCR analysis was performed using a CFX96 Touch Real‐Time PCR Detection System and components of the iQ SYBR green Supermix (Bio‐Rad). The PCR primer sequences and cycle amplification protocol are indicated in Table 2. Amplification reactions were performed in a total volume of 10 μl containing 0.5 μl cDNA, 5 μl SsoAdvanced^TM^ SYBR^®^ Green Supermix (Bio‐Rad), and 1.5 pmoL forward and reverse gene‐specific primers. The baseline was used to determine the *C*_t_ (cycle threshold) in each reaction. The melting curve was constructed for each primer pair to verify the presence of one gene‐specific peak and the absence of primer dimer. For the quantification, standard curves were generated by amplifying serial dilutions of each amplicon. For each primer pair, efficiency curves were generated using serial dilutions of cDNA (0.05–50 ng per reaction) in the abscissa and the corresponding *C*_t_ in the ordinate; all values were within acceptable limits (Livak & Schmittgen, 2001), and no primer dimers were formed. The experimental samples were indeed detected within the validated standard range. The *C*_t_ of the target gene was compared with the internal reference gene *GAPDH*. Each sample was tested in triplicate, and the mean value was used (Bustin et al., 2009).

**Table 2:** Primer sequence for real‐time PCR and condition for real‐time PCR

Genes	Sequence(5′→3′)	Accession no.	Amplicon size (bp)	Cycle profile	Cycles	PCR efficiency (%)
*FSHR*	F: AGTGACACCAAGATAGCCAAGC	KJ817181	151	95°C/10 s, 51.6°C/20 s	40	97.2
	R: GGTAGAACAGGACCAGGAGGAT					
*LHCGR*	F: TTCAATGGGACAACGCTGATTTC	KP310926	174	95°C/10 s, 50.6°C/20 s	40	94.3
	R: TGTGGCAATTAGCGTCTGAATGGA					
*ESR2*	F: GCCTCCATGATGATGTCCTTGA	EU847286	117	95°C/10 s, 49.7°C/20 s	40	99.4
	R: GAGCCGCACTTGGTCGTA					
B4GALNT2	F:TCCGCTTTCCTGTTATGCC R: CAAACCAACCCTTGCCGTAG	KC175557	240	95°C /10 s, 59.5°C /20 s, 72°C/30 s	35	98.2
BCL2	F: TCGCCCTGTGGATGACCG	KJ782301	^134^	94°C/40 s, 49.7°C/20 s	40	99.9
	R: CAGAGACAGCCAGGAGAAAT					
BAX	F: CCGAGTGGCGGCTGAAATGT	KJ782302	^161^	94°C/40 s, 51.7°C/40 s	40	97.9
	R: GCTCTCGAAGGAAGTCCAATGT					
GAPDH	F：TGCCAAGTATGATGAGAT R: GAAGGTAGAAGAGTGAGT	NC_022297	^130^	95°C;/10 s,57.6°C/20 s	40	92.2

Abbreviations: F, Forward primer; R, Reverse primer.

### 2.8 Statistical analysis

All of the data are expressed as means ± standard error of the mean (*SEM*). A *t *test was used to compare hormone concentrations and the results of qPCR quantification between the two breeds. The experiments were performed in three replicates.

## 3 RESULTS

### 3.1 Plasma hormone concentration

**Figure 1:**
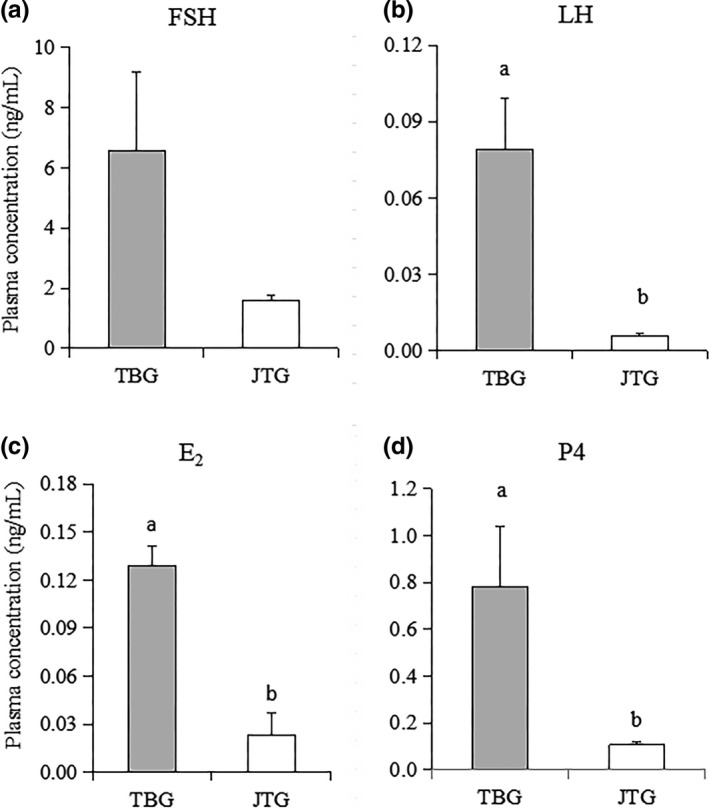
Serum hormone concentrations of Tibetan goat (TBG) and Jintang black goat (JTG). (a) FSH. (b) LH. (c) E_2_. (d) P4. Blood samples were collected by venipuncture at 40 hr after CIDR removal. Values represent the mean ± SEMs (*n* = 5 per breed). Different letters indicate the difference between breeds (*p* < .05)

The plasma hormone concentration results are summarized in Figure 1. The mean FSH concentration was more than four‐fold higher in the TBG (6.57 ± 2.64 ng/ml) than in the JTG (1.59 ± 0.35 ng/ml) group, but the difference for FSH did not reach significance, most probably due to a high variability among the TBG animals. Plasma concentrations of LH, P_4_, and E_2_ were lower in the JTG than in the TBG group (*P* ˂ 0.05).

### 3.2 Variations in nucleotide and deduced amino acid sequences

From the mRNA analysis, the open reading frames of *FSHB*, *LHB*, *FSHR*, *LHCGR*, *B4GALNT2*, *BCL2* and *BAX* were 390, 426, 2,088, 2,103, 1,521, 690 and 579 bp, respectively. All of the sequences were deposited in the GenBank database (Table 3). An analysis of the *FSHB*, *LHB*, *FSHR*, *LHCGR*, *B4GALNT2*, *BCL2* and *BAX* nucleotide sequences showed that the JTG PCR products were 99.74, 100, 99.95, 99.57, 99.60, 99.57 and 100% identical, respectively, to the TBG PCR products. The differences in nucleotide and deduced amino acid sequences between the two breeds are shown in Table 3. The sequences of the *LHB* and *BAX* genes were not different between the two breeds. There was one base change in the *FSHR* gene and three base changes in the *BCL2* gene between the two breeds, but these base changes did not result in any amino acid substitutions. However, there was one base change in the *FSHB* gene that resulted in one amino acid substitution; there were nine base changes in the *LHCGR* gene that resulted in five amino acid substitutions; there were six base changes in the *B4GALNT2* gene that resulted in four amino acid substitutions.

**Table 3:** Sequence variation in Jintang black goats (JTG) and Tibetan goats (TBG)

Gene	GenBank Acc. no. (JTG; TBG)	CDS (bp)	Amino acids	Base changes (JTG → TBG)	Amino acid changes (JTG → TBG)
FSHB	KP310922; KP310923	390	129	A344G	Q114R
LHB	KP310924; KP310925	426	141	none	none
FSHR	KJ817181; KP310921	2088	695	A144G	none
LHCGR	KP310926; KP310927	2,103	700	C340T	H114Y,
				G357A	none
				G374A	R125Q
				A399G	H137R
				A410G,	none
				A499G,	R167G
				G615A,	none
				T696C	none
				C1345T	L449F
B4GALNT2	KP723683; KP723684	1521	506	T170C	none
				G340A	R113H
				G550A	G183D
				A636T	T212S
				C674T	none
				T1345C	L448F
BCL2	KJ782301; KJ782304	690	229	G96A	none
				T128C	none
				G440A	none
BAX	KJ782302; KJ782303	579	192	none	none

### 3.3 Variations in mRNA expression levels within ovaries

**Figure 2:**
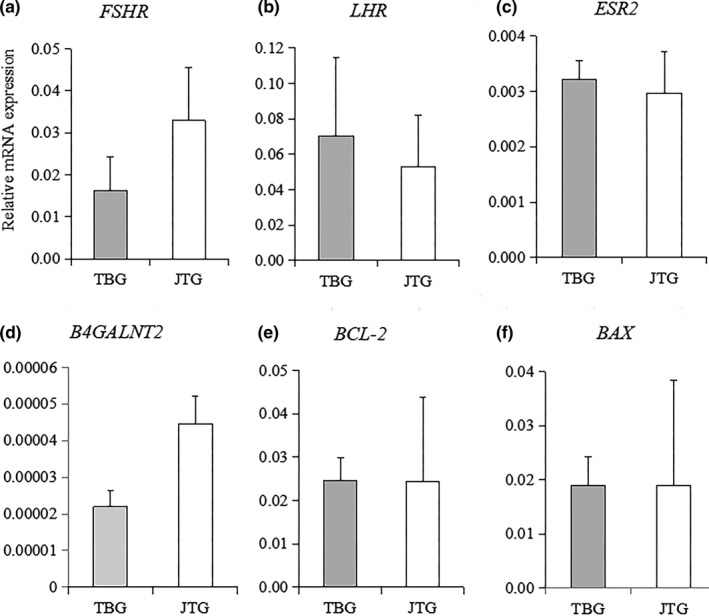
Messenger RNA expression levels of the *FSHR*, *LHCGR*, *ESR2*, *B4GALNT2*, *BCL2*, and *BAX* genes in ovaries. (a) *FSHR*. (b) *LHCGR*. (c) *ESR2*. (d) *B4GALNT2*. (e) *BCL2*. (f) *BAX*. Ovary samples were collected from Tibetan goat (TBG) and Jintang black goat (JTG) at 40 hr after CIDR removal. Values represent the mean ± SEMs (*n* = 5 per breed)

Real‐time PCR was performed to quantitate the expression of the *FSHR*, *LHCGR*, *ESR2*, *B4GALNT2*, *BCL2* and *BAX* genes in the ovaries. The results showed that the expression levels of these genes were not different between nonprolific TBG and prolific JTG at 40 hr after the CIDR removal (Figure 2).

## 4 DISCUSSION

Marked differences in ovulation rates have been found in different breeds of goats (Cui et al., 2009; Hua et al., 2008; Zi et al., 2013). This study attempted to lay a foundation for elucidating the endocrine and molecular mechanisms that control ovulation rate by comparing prolific JTG and nonprolific TBG. The onset of synchronized estrus occurred at approximately 40 hr after the CIDR withdrawal from the goats (Romano, 2004), and goats ovulated approximately 30 hr later (Menchaca, Miller, Salveraglio, & Rubianes, 2007). Therefore, we sampled at 40 hr after the CIDR removal because this is the critical period determining the dominant follicles that undergo growth or atresia.

A mutation in base 680 of the *FSHR* gene from Asn to Ser resulted in decreased FSH activity in humans (Greb et al., 2005). It has also become evident that partially inactivating mutations of FSHR can cause an arrest at the early or later stages of follicular growth (Touraine et al., 1999). Functional studies demonstrated in vitro that the nonsense mutation c.175C > T caused the loss of full‐length *FSHR* expression and that the p.R59X mutant showed no response to FSH stimulation of cAMP levels in a Chinese woman with primary ovarian insufficiency (Liu et al., 2017). The A to G mutation within the upstream region of the *FSHR* gene (position − 278) may affect some reproductive variables in Holstein dairy cows (Sharifiyazdi, Mirzaei, & Ghanaatian, 2018). The Tyr76X mutation of the FSH β‐subunit is associated with a partial phenotype of FSH deficiency in girls (Berger et al., 2005). Two mutations of g.36946470C > T and g.36933082C > T in the exon of *B4GALNT2* have a significant effect on litter size in the Small Tail Han Sheep (Guo et al., 2018). The high prolificacy of the D'man sheep is associated with the segregation of the FecLL mutation in the *B4GALNT2* gene (Ben et al., 2018). An analysis of the *FSHB*, *LHB*, *FSHR*, *LHCGR*, *B4GALNT2*, *BCL2*, and *BAX* nucleotide and amino acid sequences revealed a high degree of identity between prolific JTG and nonprolific TBG, although base changes in the *FSHR* and *BCL2* genes did not lead to any amino acid changes, and there were no differences in the *LHB* and *BAX* genes. However, base changes in the *FSHB, LHCGR*, and *B4GALNT2* genes resulted in amino acid substitutions in the translated proteins. These amino acid substitutions might induce a change in the conformation of the protein structure, thereby affecting the signaling pathway during follicle differentiation and ovulation.

Comparative studies of the endocrine profiles of sheep with or without mutations influencing ovulation rate were conducted. Prolific D’Man, Booroola, Finn ewes and Boer does have been reported to have higher FSH concentrations during the follicular phase compared with their local controls or those of nonprolific breeds (Bartlewski et al., 1999; Bindon et al., 1985; Cui et al., 2009; Lahlou‐Kassi et al., 1984). In contrast, homozygous carriers of the Booroola mutation (FecB^B^) gene and the Inverdale (FecX^I^) mutation gene do not seem to have a difference in the circulating ovarian hormones (E_2_, P_4_ and inhibin) or pituitary gonadotropins when compared to wild‐type ewes (Baird & Campbell, 1998; Campbell, Baird, Souza, & Webb, 2003; Shackell et al., 1993). Furthermore, plasma concentrations of FSH during the preovulatory period have been reported to be significantly elevated in the barely prolific Galway compared with prolific Finnish Landrace (Adams et al., 1988). In the present study, the mean FSH concentrations were not different, but the plasma concentrations of LH, P_4_ and E_2_ were lower in prolific JTG than in nonprolific TBG (*P* ˂ .05). Ovarian E_2_ normally exerts a homeostatic negative feedback on GnRH release. During the sustained exposure to elevated estradiol in the late follicular phase of the reproductive cycle, however, the feedback action of E_2_ switches to positive, inducing GnRH release from the brain, which signals pituitary LH release (Christian & Moenter, 2010). Higher ovulation rates are often accompanied by smaller ovulatory follicles and fewer granulosa cells per follicle with less estradiol production in Chios sheep (Avdi, Chemineau, & Driancourt, 1997). The plasma concentration of LH at the peak of the surge was significantly reduced in the Finnish Landrace line that was selected for its high ovulation rate (Adams et al., 1988). Thus, a significant increase in the LH level might be as a result of the increase in the E_2_ level, which may reduce the ovulation rate by shortening the follicular phase in TBGs. The administration of P_4_ at the end of diestrus decreased the incidence of ovulations from the penultimate wave of the estrous cycle (Bartlewski et al., 2017). Therefore, the higher plasma concentrations of P_4_ in TBGs might also be related to their lower ovulation rate. This observation was contrary to the results from Drouilhet et al. (2010) in the highly prolific Lacaune sheep.

It is possible that prolific and nonprolific breeds of sheep and goats are characterized by differences in their ovaries, i.e., the sensitivity of follicular cells to gonadotropins by a greater expression of gonadotropin receptors in the follicular cells of prolific breeds (Abdennebi et al., 1999; Cui et al., 2009; Drouilhet et al., 2010; Regan et al., 2015) and in their paracrine regulation (Drouilhet et al., 2013; Hunter et al., 2004). However, the differences in the mRNA expression levels of the *FSHR*, *LHCGR*, *ESR2*, and *B4GALNT2* genes in the ovaries were not noted between prolific JTG and nonprolific TBG. While there is no significant difference between breeds for any of the genes, the patterns are different. For example, the average ratio is 0.5 for *B4GALNT2* with a limited variability between individuals of a breed; the difference may become significant with a larger number of individuals (*n* ≥ 422). By contrast, *BCL2* and BAX display a similar average expression in both breeds but a large variability among JTG animals. Whole ovaries were homogenized and used for RNA purification in this study, which likely diluted any specific changes that may be present within ovarian follicles from different developmental stages. However, our previous study also showed that the expression levels of *FSHR* and *LHCGR* mRNA in the follicles of non‐prolific TBG goats were 5.1‐fold and 7.3‐fold greater, respectively, than those in the prolific Lezhi goat, and the expression level of follicle *ESR2* was not different between the prolific Lezhi black goat and nonprolific TBG (Zi et al., 2013). Cui et al. (2009) reported that the ovarian expression levels of *FSHR* were lower but that *ESR2* was higher in nonprolific Yunling black goats than that in prolific Boer goat. The reason for this discrepancy is not known, but it indicates that the mechanisms controlling ovulation rate may be different in different breeds of goats.

It has been widely accepted that mammalian females are born with a nonrenewing, finite pool of oocytes that will be continuously cleared by atresia, with only a small proportion of them reaching ovulation. Apoptosis regulates this mass germ cell death, especially through the balance between the pro‐ and anti‐apoptotic proteins that are encoded by the *BCL2* gene family. A reduced expression of *BCL2*, increased *BAX* expression and increased ratio of *BAX* to *BCL2* expression promotes the apoptosis of atretic follicles (Almog et al., 2001; Gursoy et al., 2008; Jensen et al., 2006; Sai et al., 2011; Van Nassauw et al., 1999). The natural preferential expression of BCL2 and restricted BAX expression greatly suppress apoptosis in the ovary of *L. maximus*, which prevents the decrease of the oocyte reserve by abolishing follicular atresia and enables the highest ovulation rate known for a mammal, 400–800 or more eggs per cycle (Jensen et al., 2006). However, the differences in mRNA expression levels of *BCL2* and *BAX* and the ratio of *BAX* to *BCL2* gene expression in ovaries were not noted between prolific JTG and nonprolific TBG, but a large variability was observed in the JTG animals. The difference in expression levels of these genes in the follicles from different developmental stages also needs to be further studied.

## 5 CONCLUSIONS

In conclusion, to our knowledge, this is the first study to investigate the variation in the plasma concentrations of FSH, LH, E_2_ and P_4_ and the sequences and ovarian mRNA expression levels of the *FSHR, LHCGR*, *ESR2*, *B4GALNT2*, *BCL2* and *BAX* genes between prolific Jintang black goats and nonprolific Tibetan goats. The plasma concentrations of LH, P_4_, and E_2_ were lower in the prolific breed than those in the nonprolific breed, but ovarian mRNA expression levels of the studied genes were not different between the two breeds. There were variations in the amino acid sequences of *FSHB, LHCGR* and *B4GALNT2*, but further research will be necessary to confirm whether the variations in the amino acid sequences of *FSHB, LHCGR* and *B4GALNT2* affect follicular development and atresia in goats.

## CONFLICT OF INTEREST

All authors declare no conflict of interest.

## AUTHOR CONTRIBUTIONS

Study design and manuscript preparation: XDZ, YCZ. Laboratory work: XDZ, LH, JYL, SL.

## ETHICAL STATEMENT

The authors confirm that the ethical policies of the journal, as noted on the journal's author guidelines page, have been adhered to and the appropriate ethical review committee approval has been received. The Regulations for the Administration of Affairs Concerning Experimental Animals (Ministry of Science and Technology, China; revised in August 2011) was followed.
